# Opioid use in medical cannabis authorization adult patients from 2013 to 2018: Alberta, Canada

**DOI:** 10.1186/s12889-021-10867-w

**Published:** 2021-05-01

**Authors:** Cerina Lee, Mu Lin, Karen J. B. Martins, Jason R. B. Dyck, Scott Klarenbach, Lawrence Richer, Ed Jess, John G. Hanlon, Elaine Hyshka, Dean T. Eurich

**Affiliations:** 1 School of Public Health, 2-040 Li Ka Shing Centre for Health Research Innovation, University of Alberta, 11203-87 Avenue, Edmonton, AB T6G 2E1 Canada; 2 SPOR (Strategy for Patient Oriented Research) Data Platform, Alberta Health Services, Edmonton, Alberta Canada; 3 Faculty of Medicine & Dentistry, University of Alberta, Edmonton, Alberta Canada; 4 Cardiovascular Research Centre, Department of Pediatrics, Faculty of Medicine and Dentistry, University of Alberta, Edmonton, Alberta Canada; 5 College of Physicians & Surgeons of Alberta, Edmonton, Alberta Canada; 6 St. Michael’s Hospital Department of Anesthesia, University of Toronto, Toronto, Ontario Canada; 7 Department of Anaesthesiology and Pain Medicine, University of Toronto, Toronto, Ontario Canada

**Keywords:** Opioid morphine equivalence, Opioid, Chronic pain, Medical cannabis, Epidemiology, Cohort study

## Abstract

**Background:**

The opioid overdose epidemic in Canada and the United States has become a public health crisis - with exponential increases in opioid-related morbidity and mortality. Recently, there has been an increasing body of evidence focusing on the opioid-sparing effects of medical cannabis use (reduction of opioid use and reliance), and medical cannabis as a potential alternative treatment for chronic pain. The objective of this study is to assess the effect of medical cannabis authorization on opioid use (oral morphine equivalent; OME) between 2013 and 2018 in Alberta, Canada.

**Methods:**

All adult patients defined as chronic opioid users who were authorized medical cannabis by their health care provider in Alberta, Canada from 2013 to 2018 were propensity score matched to non-authorized chronic opioid using controls. A total of 5373 medical cannabis patients were matched to controls, who were all chronic opioid users. The change in the weekly average OME of opioid drugs for medical cannabis patients relative to controls was measured. Interrupted time series (ITS) analyses was used to assess the trend change in OME during the 26 weeks (6 months) before and 52 weeks (1 year) after the authorization of medical cannabis among adult chronic opioid users.

**Results:**

Average age was 52 years and 54% were female. Patients on low dose opioids (< 50 OME) had an increase in their weekly OME per week (absolute increase of 112.1 OME, 95% CI: 104.1 to 120.3); whereas higher dose users (OME > 100), showed a significant decrease over 6 months (− 435.5, 95% CI: − 596.8 to − 274.2) compared to controls.

**Conclusions:**

This short-term study found that medical cannabis authorization showed intermediate effects on opioid use, which was dependent on initial opioid use. Greater observations of changes in OME appear to be in those patients who were on a high dosage of opioids (OME > 100); however, continued surveillance of patients utilizing both opioids and medical cannabis is warranted by clinicians to understand the long-term potential benefits and any harms of ongoing use.

**Supplementary Information:**

The online version contains supplementary material available at 10.1186/s12889-021-10867-w.

## Highlights

- Medical cannabis authorization showed mixed effects on short-term opioid utilization
- Greatest reductions in opioid use appeared to be in patients who started with a high dosage of opioids
- Ongoing evidence for clinicians is warranted regarding the potential impact of medical cannabis for pain management and as an alternative to opioid medication

## Background

For both the United States (US) and Canada, the over-prescription and widespread diversion of pharmaceutical opioids has led to significant health harms and become a major burden on the healthcare system – with a global estimate 26–36 million individuals abusing opioids [1, 2]. For centuries, opioids have been a classification of drug commonly used to manage both acute and chronic pain [3]. However, chronic opioid exposure has led to both opioid misuse and abuse [4], in particular, in the occurrence of opioid use disorder [5, 6], opioid-related deaths [7], and diversion of opioid medication to those without prescriptions [8]. In Alberta alone, the provincial quarterly opioid response surveillance system [9] has reported 449 deaths from January–June 2020; and another 284 deaths from June–September 2020. With the COVID-19 pandemic, this number reached record levels and has continued to increase (likely due to a decrease in the use of harm reduction and healthcare services). Unfortunately, these same trends exist in all provinces in Canada and in the United States.

Indeed, identifying suitable medical alternatives to opioids for both chronic and acute pain has become a critical area of investigation [10, 11], specifically to assist patients in reducing their overall opioid use and limit unnecessary exposure for opioid-naïve populations. An increasing body of literature suggests that medical cannabis may decrease chronic pain [12, 13], be a potential substitute for opioids [14] and act as a contender for decreasing patients’ opioid usage [15]. Known as the “opioid-sparing effect,” recent studies have emphasized the analgesic properties [16, 17] of medical cannabis – and that concomitant use with cannabis may potentially show a significant reduction in overall reliance of opioid usage [18] – and consequently, lead to an improved quality of life.

The best-available clinical guidelines from the US [19] report that there is substantive and/or conclusive evidence regarding cannabis effects on pain [20, 21] (in particular, improvements on neuropathic pain). Likewise, past systematic reviews [3, 22–25] concur that cannabis use has shown benefits in pain reduction. Recent observational studies [26–28] in the US further support the potential for cannabis to act as an adjunct for opioid medication. Other studies suggest that medical cannabis access laws may also contribute to the reduction of the number of opioids prescribed per year [4, 29, 30]. Despite the plethora of studies linking medical cannabis with decreased opioid use, they also highlight a major limitation in that the extent of the effectiveness of cannabis use on opioid reduction is highly variant - depending on the type of pain, type/dosage of opioid medication, and concomitant use of other medications. Importantly, the long-term effect of cannabis on opioid use is also unknown. Interestingly, Canadian guidelines [31] have taken a more cautionary approach to cannabis, recommending that although there may be a small benefit for reducing chronic pain, medical cannabis should not be utilized as a primary line of treatment for pain, if possible. Rather, expert bodies [31] emphasize that evidence needs to be supplemented with additional longitudinal studies in order to effectively validate this association. Currently, there are very few rigorous longitudinal population studies [32, 33] that study the impact of cannabis use on opioid medication.

Medical cannabis access has been legal in Canada for two decades. However, the legalization of non-medical cannabis in Canada (October 2018) and in several US states, has coincided with broader public interest in the therapeutic properties of cannabis and this includes its potential to be a treatment for pain, it is imperative to close the evidence gap on the relationship between medical cannabis on prescription opioid use. Thus, the purpose of the study was to assess the influence of medical cannabis authorization on chronic opioid use. We hypothesized that adult patients on chronic opioid treatment who are authorized to use medical cannabis would experience an opioid-sparing effect, defined as an overall decrease in oral morphine equivalence (OME) use over time compared to controls.

## Methods

### Study design

A matched cohort study among chronic opioid users authorized to use medical cannabis and controls who did not receive authorization for medical cannabis.

### Population

#### Inclusion criteria

All patients prescribed chronic opioid treatment and authorized for medical cannabis in Alberta (data received from the College of Physicians & Surgeons of Alberta which authorizes all medical use in the province) between March 22, 2013 and March 31, 2018. In Canada, medical cannabis authorization is defined as a patient being granted authorization by their health care provider to access cannabis for medical purposes. Participants were adults of any sex, ethnicity, and socioeconomic status who received authorization for medical cannabis for any reason. Chronic opioid treatment was defined as: 1) all patients who had an opioid prescription within 7 days prior to the index date (90% of opioid prescriptions in Alberta are 7 days or less), and 2) either had a total of 120 or more cumulative calendar days of filled opioid prescriptions or 10 or more opioid prescriptions filled in the year prior to the index date [34–37]. The index date for each patient was the first recorded date of medical cannabis authorization. The index date of all eligible controls was the first opioid dispensation date plus 1 year. The one-year period between the first opioid dispensation date and index date served as the wash period.

#### Exclusion criteria

All patients who received medical cannabis but were not registered to receive health benefits in Alberta during the entire study period, were excluded from the study. Patients who had less than 6 months administrative data before the index date were excluded as changes in weekly average OME could not be reliability calculated. Further, patients who had codeine cough syrups up to 1 year before the index date were excluded as codeine cough syrup is often prescribed for its antitussive properties, as opposed to pain relief.

### Propensity score matched controls

Each authorized medical cannabis patient was matched with one unique control by using high dimensional propensity score (HDPS) matching [38]. Controls had to satisfy the same inclusion and exclusion criteria as authorized medical cannabis patients – but without authorization of medical cannabis. Variables incorporated into the HDPS matching method included: sex, age, year of index date (categorical), comorbidities associated with cannabis use (Additional file 1: Tables S1 and S2), and all healthcare resource utilization variables (all within the year prior to the index date). This includes healthcare utilization (all hospitalizations, emergency department visits, physician visits with up to 25 diagnostic codes) (Additional file 1: Table S2; Additional file 2), and all prescription drug utilized by a patient. Of note, our entire healthcare dataset reported greater than 1000 different variables and categories which were included in the HDPS. Administrative data sources (see below) used as the input datasets of the HDPS included: inpatient hospital data, ambulatory visit data and claims data. Similar to those authorized to use medical cannabis, eligible controls had to be users of opioid medication between March 2012 to 2017 and the index date of each control was set to the first opioid use date + 365 days to allow stabilization of therapy. We applied the HDPS matching technique using the SAS Packages proposed by Rassen et al. [39] and Schneeweiss et al. [38].

### Data source

The initial medical cannabis patient identifiers were provided by the College of Physicians & Surgeons of Alberta. Using a unique lifetime personnel health number, all patients were linked to the administrative databases of Alberta Health which captures all healthcare utilization for all patients in the province of Alberta as part of the universal healthcare plan for residents. These databases include all inpatient hospitalizations, ambulatory emergence department visits, all community pharmacy drug dispensations, and physician claims data, providing at least one-year of longitudinal follow-up data following the index date for both medical cannabis authorized patients and controls. All data was released as de-identified data to the researchers.

### Outcomes

All opioid doses were converted to OME based on each drug’s OME factor, days of supply, dispensation amount and strength. Each daily OME was then converted to a weekly average OME for all medical cannabis patients and matched controls [40, 41] for each week of the study based on the index date. The primary outcome was the difference in the weekly average OME between the medically authorized patients and the control group in the 6 months prior to index and up to 12 months following medical cannabis authorization (or equivalent index date for controls). The average weekly OME in the 6 months prior to authorization (or pseudo index for the matched control) was used as the baseline.

A secondary outcome was the “proportion of patients ceasing opioids”, defined as opioid discontinuation after the index date for 2 distinct circumstances: 1) opioid-free for twice the duration of the previous prescription or; 2) a minimum grace period of 30 opioid-free days. As the duration of opioid prescriptions have been known to be highly variable, we utilized this specific protocol following the guidance of Gomes et al. [33], although over 90% of all opioid dispensations in Alberta are < 7 days.

### Ethics approval

This study was approved by the University of Alberta Health Research Ethics board (PRO 00084689).

### Statistical analysis

All data are expressed descriptively using means (standard deviations [SD]) or count (proportions [%]), as appropriate. To assess the effect of medical cannabis use on weekly average OME, interrupted time series (ITS) analysis was used to assess the change in trend of OME in the 26 weeks (6 months) before and 52 weeks (1 year) after the authorization of medical cannabis (or pseudo index for the matched control). ITS is a quasi-experimental design that allows comparison of trends in an outcome before and after an intervention [42, 43]. ITS analysis was selected for its effectiveness in clear differentiation between population-level health pre-intervention and post-intervention periods. The ITS analysis allows cannabis patients to be compared to themselves (i.e. their own control) by modeling their OME trend in the 52 weeks (1 year) after the authorization of medical cannabis relative to the trend they had in the 26 weeks before. However, the basic interrupted time series design cannot exclude confounding due to temporal changes at the population level around the time of the intervention (i.e., cannabis authorization) such as co-interventions. The controlled ITS, which we employed, includes an additional control series to account for temporal changes that may have occurred within the population. Indeed, the controlled interrupted time series [44] has been shown to provide similar results as those observed in RCTs [45, 46], a testament to the validity of the approach [47, 48].

OME was assessed in 7-day windows for each patient (i.e. average OME per week). The absolute effect of medical cannabis authorization on average weekly OME was calculated, which summarizes both the immediate level change (i.e., within a week) and change in trend over the 12 months with the multivariate delta method used to the construct 95% confidence intervals around the estimate [49].

To assess the proportion of patients ceasing opioids after the index date, a logistic regression model was used to compare the odds of opioid discontinuation after medical cannabis authorization between the authorized and unauthorized patients.

### Sensitivity and stratification analysis

Further stratification was conducted on both authorized medical cannabis patients (*n* = 5373) and all eligible control (*n* = 24,693) patients in 3 subgroups of baseline average OME (based on an average over all 26 weeks before index date): i) OME ≤ 50, ii) OME between 50 and 100, and iii) OME > 100. Patients were matched within each category on an average weekly OME ±15.

## Results

**Fig. 1:**
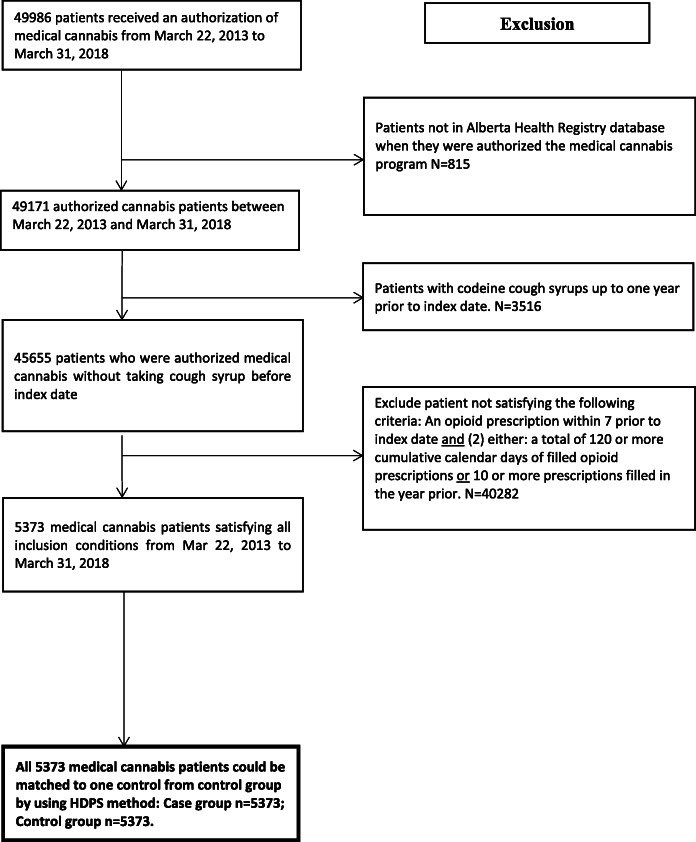
Selection of study population

In total, 5373 medically authorized cannabis patients and 24,693 eligible controls were identified (Fig. 1) with differences noted between the groups (Additional file 1: Table S1). All 5373 patients were matched to one control and following HDPS matching, and all covariates were well balanced after matching between the groups (standardized differences < 10%; a threshold often recommended for declaring imbalance in pharmacoepidemiological research [50]) (Table 1).

**Table 1:** Baseline characteristics of those authorized for medical cannabis and matched controls (*n* = 10,746)

Characteristic	Matched Controls (***N*** = 5373)	Authorized for medical cannabis (***N*** = 5373)	***P***-value	Standardized Difference
Age, years, mean (SD)	52.5 (15.8)	52.3 (13.9)	0.74	0.00638
Female, n (%)	2948 (54.9%)	2907 (54.1%)	0.70	0.00638
**Comorbidities**				
Neoplasms, n (%)	1039 (19.3%)	1117 (20.8%)	0.06	0.03626
Diabetes, n (%)	838 (15.6%)	830 (15.5%)	0.83	0.00411
Mental Disorder, n (%)	3832 (71.3%)	3857 (71.2%)	0.59	0.01031
Nerve System Disease, n (%)	1391 (25.9%)	1536 (28.6%)	0.01	0.06065
Chronic Obstructive Pulmonary Disease, n (%)	985 (18.3%)	921 (17.1%)	0.11	0.03119
Colitis, n (%)	174 (2.2%)	159 (3.0%)	0.01	0.04943
Inflammatory Disease of Uterus, n (%)	3 (0.1%)	3 (0.1%)	1.0	0
Diseases of the Musculoskeletal System and Connective Tissue, n (%)	4605 (85.7%)	4703 (87.5%)	0.01	0.05359
Generalized Pain, n (%)	5 (0.1%)	8 (0.2%)	0.40	0.01606
Injury and Poisoning, n (%)	1838 (34.2%)	1686 (31.4%)	0.01	0.06029
**Healthcare Utilization**				
Patients with at least one inpatient hospitalization, n (%)	1062 (19.7%)	1058 (19.7%)	0.92	0.00187
Patients with at least five outpatient visits, n (%)	2271 (42.3%)	2280 (42.4%)	0.86	0.00339
Patients with at least five distinct drug class dispensations, n (%)	5231 (97.4%)	5181 (96.4%)	0.01	0.05364

**Fig. 2:**
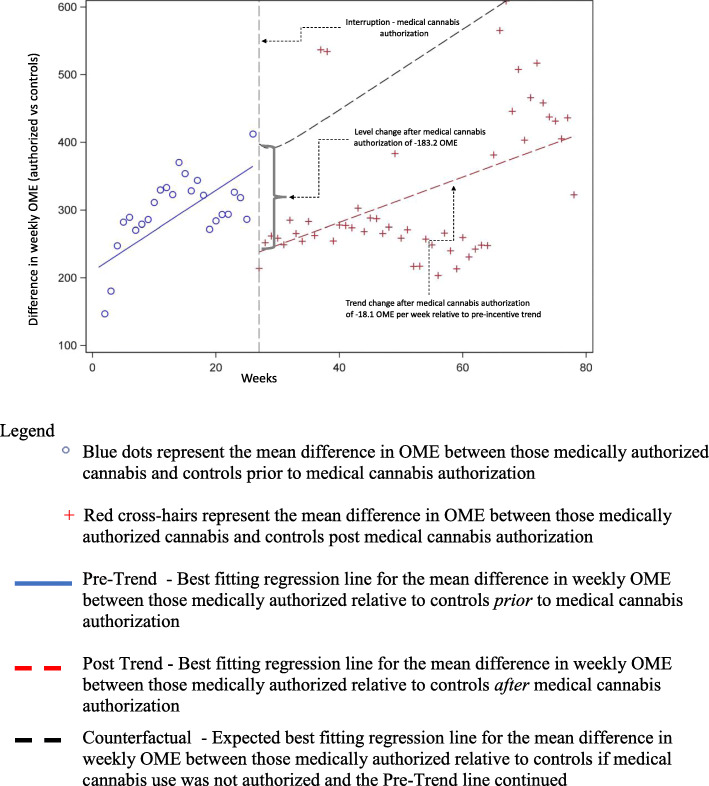
Difference in mean weekly oral morphine equivalents per patient for medically authorized cannabis users (*n* = 5373) vs matched controls (*n* = 5373)

Over the 52 week follow-up period after medical cannabis authorization, there was an initial decrease in the weekly average OME use in authorized medical cannabis patients in comparison to matched controls (− 183.2 OME, 95% CI: − 449.8 to 83.3) per patient (Table 2, Fig. 2), although it was not statistically significant. However, there was a consistent significant decrease in the week-to-week trend change after cannabis authorization (− 18.1 OME, 95% CI: − 29.1 to − 7.2) per patient relative to controls. Combined, there was a non-significant decrease in the absolute difference in the total weekly OME (− 76.5, 95% CI: − 308.0 to 154.9) per patient between cases and controls (Table 2).

**Table 2:** Interrupted time series of mean weekly OME differences per patient in medically authorized cannabis users (*n* = 5373) vs controls (*n* = 5373)

Variable	Weekly OME difference (95% CI)^**a**^
Pre-incentive trend^b^	18.7 (11.2 to 26.3)
Level change after medical cannabis authorization^c^	−183.2 (−449.8 to 83.3)
Trend change after medical cannabis authorization^d^	−18.1 (− 29.1 to − 7.2)
Overall absolute effect after medical cannabis authorization^e^	−76.5 (− 308.0 to 154.9)

^a^All reported values indicate the average difference in weekly mean OME per patient in those who received a medical cannabis authorization compared to controls

^b^Rate of change in the outcome over time prior to medical cannabis authorization

^c^Immediate change in outcome following medical cannabis authorization

^d^week to week change in mean OME or slope after medical cannabis authorization, relative to the pre-incentive difference in trend

^e^The overall absolute effect after medical cannabis authorization is the absolute difference in the weekly OME over the 26 weeks pre- and 52 weeks post-medical cannabis authorization period, compared to the counterfactual difference in trends had medical cannabis authorization not occurred (i.e. pre-incentive difference in trends projected forward)

With respect to prescription opioid discontinuation any time after the index date, overall 49.3% (2650/5373) and 72.3% (3887/5373) of those authorized medical cannabis and controls, respectively, ceased opioid dispensation during the follow-up (*p* < 0.001). Medically authorized cannabis patients were less likely to completely cease opioid medication use relative to controls; adjusted odds ratio 0.38 (0.34, 0.41) (Table 3).

**Table 3:** Unadjusted logistic regression estimates of the odds ratio to cease opioids in medically authorized cannabis users (*n* = 5373) vs matched controls^a^ (*n* = 5373)

Model variables	OR (95% CI)^a^
Exposed vs Unexposed	0.38 (0.34, 0.41)

^a^Controls served as the reference group

### Sensitivity analyses results

Among patients consuming ≤50 OME, there were minimal decreases immediately after cannabis authorization compared to controls in weekly average OME use (level change, − 1.14 OME, 95% CI: − 1.76 to − 0.52) per patient. This was followed by a slight increase in the week-to-week trend change (5.8 OME, 95% CI: 5.53 to 6.11) per patient. Overall, there was a significant increase in the absolute difference in the total weekly OME among medically authorized patients after accounting for both the immediate and overall trend during following (112.1 OME, 95% CI: 104.1 to 120.3) (Table 4) compared to controls.

**Table 4:** Interrupted time series estimates of mean weekly OME differences within baseline OME subgroups per patient in medically authorized cannabis users (*n* = 5373) vs controls (*n* = 5373)

Variable	Weekly OME difference in those < =50 OME (***n*** = 2821 cannabis patients)^**a**^	Weekly OME difference in those 50–100 OME (***n*** = 807 cannabis patients)^**a**^	Weekly OME difference in those > 100 OME (***n*** = 1103 cannabis patients)^**a**^
Pre-incentive trend^b^	−0.05 (−0.07 to −0.04)	− 0.09 (− 0.22 to 0.03)	4.4 (2.9 to 5.9)
Level change after medical cannabis authorization^c^	−1.14 (− 1.76 to − 0.52)	−17.8 (−31.8 to −3.9)	189.9 (93.2 to 286.5)
Trend change after medical cannabis authorization^d^	5.8 (5.53 to 6.11)	− 0.06 (− 0.40 to 0.29)	− 12.6 (− 15.0 to −10.2)
Overall absolute effect after medical cannabis authorization^e^	112.1 (104.1 to 120.3)	− 17.8 (− 27.3 to − 8.3)	−435.5 (− 596.8 to − 274.2)

^a^All reported values indicate the average difference in weekly mean OME per patient in those who received a medical cannabis authorization compared to controls

^b^Rate of change in the outcome over time prior to medical cannabis authorization

^c^Immediate change in outcome following medical cannabis authorization

^d^week to week change in mean OME or slope after medical cannabis authorization, relative to the pre-incentive difference in trend

^e^The overall absolute effect after medical cannabis authorization is the absolute difference in the weekly OME over the 26 weeks pre- and 52 weeks post-medical cannabis authorization period, compared to the counterfactual difference in trends had medical cannabis authorization not occurred (i.e. pre-incentive difference in trends projected forward)

Among patients consuming 50 to 100 OME, a small immediate decrease after cannabis authorization in the weekly average OME use compared to controls was observed (− 17.8 OME, 95% CI: − 31.8 to − 3.9) per patients. There were minimal changes in the week-to-week trend change over the follow-up (− 0.06 OME, 95% CI: − 0.40 to 0.29). Overall, a decrease in the absolute difference in the total weekly OME (− 17.8 OME, 95% CI: − 27.3 to − 8.3) per patient was observed (Table 4).

Lastly, among patients using > 100 OME, there was a significant immediate increase after cannabis authorization in the weekly average OME compared to controls (189.9 OME, 95% CI: 93.2 to 286.5). However, this increase was followed by a decrease in the week-to-week trend change (− 12.6 OME, 95% CI: − 15.0 to − 10.2) per patient for those authorized cannabis compared to controls. Overall, there was a significant drop in the absolute difference in the total weekly OME (− 435.5 OME, 95% CI: − 596.8 to − 274.2) per patient (Table 4).

Similar to the overall findings, irrespective of subgroup, those authorized medical cannabis were less likely to completely cease prescription opioid consumption relative to controls (Table 5).

**Table 5:** Logistic regression estimates of the odds ratio to cease opioids in medically authorized cannabis user subgroups vs controls

Baseline OME	OR (95% CI)	Number of patients in each sub-group		Proportion of cease opioid after index date, n (%), in each sub-group	
		Control	Authorized	Control	Authorized
**OME ≤ 50**	0.55 (0.49, 0.62)	2821	2821	2073 (73.5%)	1710 (60.6%)
**50 < OME ≤ 100**	0.46 (0.38, 0.56)	807	807	485 (60.1%)	331 (41.0%)
**OME > 100**	0.34 (0.28, 0.42)	1103	1103	635 (57.7%)	360 (32.6%)

## Discussion

This short-term analysis on this population-based study of patients in Alberta, Canada, showed that authorization for medical cannabis had intermediate effects on weekly OME in adults prescribed chronic opioids treatment, which was dependent on initial opioid dose. Overall, there were a range of OME reductions that were observed for all patients – with the majority of these reductions being of less clinical importance and/or non-significant. For those prescribed < 100 OME per week, no statistically significant decreases were observed in weekly OME. It is important to consider, that the majority of patients authorized medical cannabis in our study were consuming < 100 OME per week. Thus, for these patients (largest subgroup), it is uncertain how clinically important the improvement in overall opioid use is, as overall effects were relatively small at the population level. There is potential that some of these patients may have reduced their OME consumption over a longer time frame, however, this cannot be determined in this study.

Among those prescribed high doses of opioids (OME > 100), there were significant reductions in opioid consumption. It is unclear what change in weekly OME would be considered as important, but for the purposes of this study, we considered a total reduction of > 400 OME among patients prescribed high doses to be potentially clinically important. Thus, specific to AB patients, we considered these individual-level reductions to have important implications for Albertans who are currently chronic opioid users and considering medical cannabis use to decrease their opioid use. Lastly, those authorized medical cannabis were less likely to completely cease prescription opioids compared to controls, although this may not be unreasonable given the short-term follow-up in our study.

The fact that patients consuming lower doses of opioids (largest group out of the three groups) did not have substantial reductions in use may not be surprising as these patients may experience ‘floor effects’ whereby minimal changes in opioid use can occur as their weekly OME, and therefore daily OME, is already very low. More difficult to explain is the sudden increase in OME for those taking > 100 OME after authorization for medical cannabis; this may signal that the therapeutic benefit from medical cannabis may not be applicable at this level of opioid use. In comparison to those unauthorized for medical cannabis, on average, medical cannabis users typically have higher co-morbidities, disease burden and chronic pain [51]. Knowing this, although patients were well matched using HDPS, the initial significant increase in OME following medical cannabis authorization may be due to the fact that opioid use may be higher because this subset of patients are more likely to have severe chronic pain and were prescribed a higher short-term opioid dose to control pain while cannabis was initiated. Given the nature of the study design, it is impossible to determine the true underlying cause of the initial increase. Regardless, our findings showed a consistent week-to-week trend (and ongoing) of a significant decrease in OME use over time for those using > 100 OME per week.

Comparatively, our findings align with the clinical recommendations from the United States on the use of medical cannabis for chronic pain; however, they do not contribute to evidence regarding medical cannabis’ effectiveness on chronic pain [19]. The most recent systematic review on medical cannabis’ impact on non-cancer pain also suggest that, despite the viability of medical cannabis for reducing opioid use, medical cannabis still cannot be considered as an adjunct treatment option to opioids [52]. Conversely, from the Canadian standpoint, Allan et al. [31] state that there is only some evidence for medical cannabis’ therapeutic benefits, and that it is limited to neuropathic pain, palliative and end-of-life pain. In fact, Canadian clinicians strongly recommend against medical cannabis (particularly smoked) as the primary avenue of pain treatment. As our study is specific to Canada, our findings may contribute new evidence and potentially clarify the population health impacts of medical cannabis use. Although this study cannot exactly quantify population-level effects of opioid reduction from medical cannabis use, what we do know is that the opioid epidemic in North America is growing. Thus, for the purposes of the study, we can infer that any type of decrease in overall opioid use can be an indicator of a potential beneficial impact for the Canadian population from the perspective of opioid use.

Collectively, there has been a growing body of literature acknowledging medical cannabis’ therapeutic analgesic properties and its potential clinical association with pain reduction [13, 14, 23], however, gaps remain in the evidence. Takakuwa et al. [26] reported that cannabis was effective as an alternative to opioids in over 60% of patients (out of 180) – however, this outcome did not predict whether the individuals eventually stopped taking opioids altogether. Reiman et al. [21] also reported that medical cannabis was a viable substitute for opioids, although their study did not have a comparison group for opioid users alone. Despite the consensus on some benefit of medical cannabis on pain reduction, there are still numerous research studies that show mixed results, emphasizing that the association is not consistently strong. In fact, Olfson et al. [27] reported that cannabis use had the opposite effect – the risk for opioid use disorder was higher in cannabis users. Further, Rogers et al. [2] stated that potential polysubstance use of other illegal substances taken for the treatment of pain was a significant limitation for fully understanding the cannabis-opioid relationship. Likewise, there are a limited number of Canadian-specific epidemiological studies [6, 53, 54] that studied medical cannabis – and even smaller numbers that studied its association with opioid use [6, 30]. The majority of these studies have very small cohort numbers and are highly reliant on self-reported outcomes. To highlight, Purcell et al. [30] reported that 45.2% of their patients successfully discontinued their opioid use – but their study was conducted in a population primarily using benzodiazepines and only had 146 patients. In all, it is apparent that ongoing long-term epidemiological studies, such as this one, are critically needed to comprehend its exact interaction with opioid use. Ideally, well-controlled clinical trials are urgently needed to be able to fully elucidate cannabis’s potential benefits with respect to opioid sparing effects in patients.

The major strength of this study is that it is currently, to our knowledge, the largest and longest population-based study of medical cannabis users in Canada that utilizes rigorous measures to track medical cannabis use with current opioid use. However, our study has limitations that should be noted. First, it is an observational study, which may lead to potential spectrum bias since our cohort of patients were those who individually sought medical cannabis for treatment. Second, there may be uncertainty as to whether the medical cannabis authorized was consumed as prescribed, and if patients elected to use alternative treatments for their pain symptoms/management. Third, given wide variability of the type of cannabis products or cannabis cultivars used, we cannot pinpoint one specific strain or dose of medical cannabis that may have attributed to the significant reduction on opioid usage or type of pain. Finally, our study is limited by the lack of clinical details of medical cannabis, any concomitant use with other non-prescription opioid or other drugs, and lastly, the exact time of onset of pain symptoms for each patient.

## Conclusions

This study found that medical cannabis authorization showed intermediate effects on opioid use, with the majority of patients on OME < 100 showing minimal decreases in OME use over time. The greatest reductions appear to be in those patients who were prescribed high dose of opioids (OME > 100). Overall, our findings may contribute ongoing evidence for clinicians regarding the potential impact of medical cannabis to reduce the opioid burden among patients. Although the clinical importance of these reductions is unclear, any reduction in opioid use may be important in the ongoing struggle to contain the opioid crisis in North America.

## Supplementary Information

**Additional file 1: Table S1.** Baseline characteristics of medically authorized cannabis patients (*n* = 5373) and all opioid controls (*n* = 24,693) prior to HDPS Matching. **Table S2.** Health Conditions and ICD-9 Codes defining Comorbidities Present in Opioid Users.

**Additional file 2.** Health Conditions and ICD-10 Codes defining Comorbidities.

## Data Availability

The data that support the findings of this study are available from the College of Physicians & Surgeons of Alberta (CPSA) and the Alberta SPOR SUPPORT Unit (https://absporu.ca). We had full permission to use this data, however, restrictions apply to the public availability of these data, which are under data access agreements for the current study.

## References

[1] Wiese B, Wilson-Poe AR (2018). Emerging evidence for cannabis’ role in opioid use disorder. Cannabis Cannabinoid Res.

[2] Rogers AH, Bakhshaie J, Buckner JD, Orr MF, Paulus DJ, Ditre JW, Zvolensky MJ (2019). Opioid and cannabis co-use among adults with chronic pain: relations to substance misuse, mental health, and pain experience. J Addict Med.

[3] Hill KP, Palastro MD, Johnson B, Ditre JW (2017). Cannabis and pain: a clinical review. Cannabis Cannabinoid Res.

[4] Lucas P, Walsh Z (2017). Medical cannabis access, use, and substitution for prescription opioids and other substances: a survey of authorized medical cannabis patients. Int J Drug Policy.

[5] Feingold D, Goor-Aryeh I, Bril S, Delayahu Y, Lev-Ran S (2017). Problematic use of prescription opioids and medicinal cannabis among patients suffering from chronic pain. Pain Med.

[6] Franklyn AM, Eibl JK, Gauthier GJ, Marsh DC (2017). The impact of cannabis use on patients enrolled in opioid agonist therapy in Ontario, Canada. PLoS One.

[7] Bradford AC, Bradford WD, Abraham A, Bagwell Adams G (2018). Association between US state medical cannabis laws and opioid prescribing in the Medicare Part D population. JAMA Intern Med.

[8] Vyas MB, LeBaron VT, Gilson AM (2018). The use of cannabis in response to the opioid crisis: a review of the literature. Nurs Outlook.

[9] Services AH (2020). Alberta COVID-19 opioid response surveillance report: Q2 2020.

[10] Choo EK, Feldstein Ewing SW, Lovejoy TI (2016). Opioids out, cannabis in: negotiating the unknowns in patient care for chronic pain. JAMA.

[11] Effiong A, Kumari P, Iqbal DS (2018). Medical marijuana applications in pain management and healthcare: the need for evidence-informed policies and not undue justice. Ann Palliat Med.

[12] Campbell G, Hall WD, Peacock A, Lintzeris N, Bruno R, Larance B, Nielsen S, Cohen M, Chan G, Mattick RP, Blyth F, Shanahan M, Dobbins T, Farrell M, Degenhardt L (2018). Effect of cannabis use in people with chronic non-cancer pain prescribed opioids: findings from a 4-year prospective cohort study. Lancet Public Health.

[13] Clem SN, Bigand TL, Wilson M (2020). Cannabis use motivations among adults prescribed opioids for pain versus opioid addiction. Pain Manag Nurs.

[14] Boehnke KF, Litinas E, Clauw DJ (2016). Medical cannabis use is associated with decreased opiate medication use in a retrospective cross-sectional survey of patients with chronic pain. J Pain.

[15] Livingston MD, Barnett TE, Delcher C, Wagenaar AC (2017). Recreational cannabis legalization and opioid-related deaths in Colorado, 2000-2015. Am J Public Health.

[16] Kosiba JD, Maisto SA, Ditre JW (2019). Patient-reported use of medical cannabis for pain, anxiety, and depression symptoms: systematic review and meta-analysis. Soc Sci Med.

[17] Shah A, Craner J, Cunningham JL (2017). Medical cannabis use among patients with chronic pain in an interdisciplinary pain rehabilitation program: characterization and treatment outcomes. J Subst Abus Treat.

[18] Romero-Sandoval EA, Fincham JE, Kolano AL, Sharpe BN, Alvarado-Vazquez PA (2018). Cannabis for chronic pain: challenges and considerations. Pharmacotherapy.

[19] The Health Effects of Cannabis and Cannabinoids (2017). The current state of evidence and recommendations for research.

[20] Deshpande A, Mailis-Gagnon A, Zoheiry N, Lakha SF (2015). Efficacy and adverse effects of medical marijuana for chronic noncancer pain: systematic review of randomized controlled trials. Can Fam Physician.

[21] Reiman A, Welty M, Solomon P (2017). Cannabis as a substitute for opioid-based pain medication: patient self-report. Cannabis Cannabinoid Res.

[22] Nielsen S, Sabioni P, Trigo JM, Ware MA, Betz-Stablein BD, Murnion B, Lintzeris N, Khor KE, Farrell M, Smith A, le Foll B (2017). Opioid-sparing effect of cannabinoids: a systematic review and meta-analysis. Neuropsychopharmacology.

[23] Campbell G, Stockings E, Nielsen S (2019). Understanding the evidence for medical cannabis and cannabis-based medicines for the treatment of chronic non-cancer pain. Eur Arch Psychiatry Clin Neurosci.

[24] Jensen B, Chen J, Furnish T, Wallace M (2015). Medical marijuana and chronic pain: a review of basic science and clinical evidence. Curr Pain Headache Rep.

[25] Koppel BS, Brust JC, Fife T, Bronstein J, Youssof S, Gronseth G (2014). Systematic review: efficacy and safety of medical marijuana in selected neurologic disorders: report of the Guideline Development Subcommittee of the American Academy of Neurology. Neurology.

[26] Takakuwa KM, Hergenrather JY, Shofer FS, Schears RM (2020). The impact of medical cannabis on intermittent and chronic opioid users with back pain: how cannabis diminished prescription opioid usage. Cannabis Cannabinoid Res.

[27] Olfson M, Wall MM, Liu SM, Blanco C (2018). Cannabis use and risk of prescription opioid use disorder in the United States. Am J Psychiatry.

[28] Nugent SM, Yarborough BJ, Smith NX, Dobscha SK, Deyo RA, Green CA, Morasco BJ (2018). Patterns and correlates of medical cannabis use for pain among patients prescribed long-term opioid therapy. Gen Hosp Psychiatry.

[29] McMichael BJ, Van Horn RL, Viscusi WK (2020). The impact of cannabis access laws on opioid prescribing. J Health Econ.

[30] Purcell C, Davis A, Moolman N, Taylor SM (2019). Reduction of benzodiazepine use in patients prescribed medical cannabis. Cannabis Cannabinoid Res.

[31] Allan GM, Ramji J, Perry D, Ton J, Beahm NP, Crisp N, Dockrill B, Dubin RE, Findlay T, Kirkwood J, Fleming M, Makus K, Zhu X, Korownyk C, Kolber MR, McCormack J, Nickel S, Noël G, Lindblad AJ (2018). Simplified guideline for prescribing medical cannabinoids in primary care. Can Fam Physician.

[32] Socias ME, Wood E, Lake S, Nolan S, Fairbairn N, Hayashi K (2018). High-intensity cannabis use is associated with retention in opioid agonist treatment: a longitudinal analysis. Addiction.

[33] Gomes T, Khuu W, Martins D, Tadrous M, Mamdani MM, Paterson JM (2018). Contributions of prescribed and non-prescribed opioids to opioid related deaths: population based cohort study in Ontario, Canada. BMJ.

[34] Wang HT, Hill AD, Gomes T, Wijeysundera DN, Pinto R, Scales DC, Fowler R, Wunsch H (2018). Opioid use after ICU admission among elderly chronic opioid users in Ontario: a population-based cohort study. Crit Care Med.

[35] Von Korff M, Saunders K, Thomas Ray G, Boudreau D, Campbell C, Merrill J (2008). De facto long-term opioid therapy for noncancer pain. Clin J Pain.

[36] Raebel MA, Newcomer SR, Reifler LM, Boudreau D, Elliott TE, DeBar L, Ahmed A, Pawloski PA, Fisher D, Donahoo WT, Bayliss EA (2013). Chronic use of opioid medications before and after bariatric surgery. JAMA.

[37] Sun EC, Darnall BD, Baker LC, Mackey S (2016). Incidence of and risk factors for chronic opioid use among opioid-naive patients in the postoperative period. JAMA Intern Med.

[38] Schneeweiss S, Rassen JA, Glynn RJ, Avorn J, Mogun H, Brookhart MA (2009). High-dimensional propensity score adjustment in studies of treatment effects using health care claims data. Epidemiology.

[39] Rassen JA, Glynn RJ, Brookhart MA, Schneeweiss S (2011). Covariate selection in high-dimensional propensity score analyses of treatment effects in small samples. Am J Epidemiol.

[40] Sharma V, Weir D, Samanani S, Simpson SH, Gilani F, Jess E, Eurich DT (2019). Characterisation of concurrent use of prescription opioids and benzodiazepine/Z-drugs in Alberta, Canada: a population-based study. BMJ Open.

[41] College of Physicians and Surgeons of Alberta. OME and DDD conversion factors. Available: http://www.cpsa.ca/wpcontent/uploads/2017/06/OME-and-DDD-Conversion-Factors.pdf.

[42] Bernal JL, Cummins S, Gasparrini A (2017). Interrupted time series regression for the evaluation of public health interventions: a tutorial. Int J Epidemiol.

[43] Hamilton I, Lloyd C, Hewitt C, Godfrey C (2014). Effect of reclassification of cannabis on hospital admissions for cannabis psychosis: a time series analysis. Int J Drug Policy.

[44] Lopez Bernal J, Cummins S, Gasparrini A (2018). The use of controls in interrupted time series studies of public health interventions. Int J Epidemiol.

[45] St. Clair T, Cook TD, Hallberg K (2014). Examining the internal validity and statistical precision of the comparative interrupted time series design by comparison with a randomized experiment. Am J Eval.

[46] St. Clair T, Hallberg K, Cook TD (2016). The validity and precision of the comparative interrupted time-series design: three within-study comparisons. J Educ Behav Stat.

[47] Fretheim A, Soumerai SB, Zhang F, Oxman AD, Ross-Degnan D (2013). Interrupted time-series analysis yielded an effect estimate concordant with the cluster-randomized controlled trial result. J Clin Epidemiol.

[48] Fretheim A, Zhang F, Ross-Degnan D, Oxman AD, Cheyne H, Foy R, Goodacre S, Herrin J, Kerse N, McKinlay RJ, Wright A, Soumerai SB (2015). A reanalysis of cluster randomized trials showed interrupted time-series studies were valuable in health system evaluation. J Clin Epidemiol.

[49] Zhang F, Wagner AK, Soumerai SB, Ross-Degnan D (2009). Methods for estimating confidence intervals in interrupted time series analyses of health interventions. J Clin Epidemiol.

[50] Stuart EA, Lee BK, Leacy FP (2013). Prognostic score-based balance measures can be a useful diagnostic for propensity score methods in comparative effectiveness research. J Clin Epidemiol.

[51] Bachhuber MA, Arnsten JH, Cunningham CO, Sohler N (2018). Does medical cannabis use increase or decrease the use of opioid analgesics and other prescription drugs?. J Addict Med.

[52] Okusanya BO, Asaolu IO, Ehiri JE, Kimaru LJ, Okechukwu A, Rosales C (2020). Medical cannabis for the reduction of opioid dosage in the treatment of non-cancer chronic pain: a systematic review. Syst Rev.

[53] Smith JM, Mader J, Szeto ACH, Arria AM, Winters KC, Wilkes TCR (2019). Cannabis use for medicinal purposes among Canadian university students. Can J Psychiatr.

[54] Turna J, Simpson W, Patterson B, Lucas P, Van Ameringen M (2019). Cannabis use behaviors and prevalence of anxiety and depressive symptoms in a cohort of Canadian medicinal cannabis users. J Psychiatr Res.

