# Supplementary material for: Opioid use in medical cannabis authorization adult patients from 2013 to 2018: Alberta, Canada
Source: BMC Public Health. 2021 May 1;21:843. doi: 10.1186/s12889-021-10867-w (PMC8088205; doi:10.1186/s12889-021-10867-w)
Supplement: Supplementary file 1 — Additional file 1: Table S1. Baseline characteristics of medically authorized cannabis patients (n = 5373) and all opioid controls (n = 24,693) prior to HDPS Matching. Table S2. Health Conditions and ICD-9 Codes defining Comorbidities Present in Opioid Users. [file 12889_2021_10867_MOESM1_ESM.docx]

**Additional file 1**

**Table S1: Baseline characteristics of medically authorized cannabis patients (n=5373) and all opioid controls (n=24693) prior to HDPS Matching**

| **Characteristic** | **Unauthorized for medical cannabis**  **(N=24693)** | **Authorized for medical cannabis**  **(N=5373)** | **p-value** | **Standardized**  **Difference** |
| --- | --- | --- | --- | --- |
| Age, years, mean (SD) | 55.6 (18.3) | 52.3 (13.9) | <0.01 | 0.1898 |
| Female, n (%) | 12431 (50.3%) | 2907 (54.1%) | <0.01 | 0.20570 |
| **Comorbidities** | | | | |
| Neoplasms, n (%) | 4332 (17.5%) | 1117 (20.8%) | <0.01 | 0.08253 |
| Diabetes, n (%) | 3865 (15.7%) | 830 (15.5%) | 0.70 | 0.00565 |
| Mental Disorder, n (%) | 14111 (57.2%) | 3857 (71.8%) | <0.01 | 0.30950 |
| Nerve System Disease, n (%) | 3596 (14.6%) | 1536 (28.6%) | <0.01 | 0.34601 |
| COPD, n (%) | 3773 (15.3%) | 921 (17.1%) | <0.01 | 0.05053 |
| Colitis, n (%) | 372 (1.5%) | 159 (3.0%) | <0.01 | 0.09844 |
| Inflammatory Disease of Uterus, n (%) | 15 (0.1%) | 3 (0.1%) | 0.88 | 0.00203 |
| Diseases of the Musculoskeletal System and Connective Tissue, n (%) | 17856 (72.3%) | 4703 (87.5%) | <0.01 | 0.38694 |
| Generalized Pain, n (%) | 20 (0.1%) | 8 (0.2%) | 0.14 | 0.02004 |
| Injury and Poisoning, n (%) | 7310 (29.6%) | 1686 (31.3%) | 0.01 | 0.03858 |
| **Healthcare Utilization** | | | | |
| Patients had at least one inpatient hospitalization, n (%) | 5641 (22.8%) | 1058 (19.7%) | <0.01 | 0.07712 |
| Patients had at least five outpatient visit, n (%) | 9133 (37.0%) | 2280 (42.4%) | <0.01 | 0.11152 |
| Patients had at least five distinct drug dispensations, n (%) | 22869 (92.6%) | 5181 (96.4%) | <0.01 | 0.16814 |

**Table S2: Health Conditions and ICD-9 Codes defining Comorbidities Present in Opioid Users**

| **Disease/Injury** | **ICD-9 Code** |
| --- | --- |
| Neoplasms | 140-239 |
| Secondary Diabetes Mellitus | 249 |
| Diabetes Mellitus | 250 |
| Mental Disorders |  |
| Psychoses | 290-299 |
| Alcohol Induced Mental Disorders | 291 |
| Drug-induced Mental Disorders | 292 |
| Schizophrenic Disorders | 295 |
| Episodic Mood Disorders | 296 |
| Delusional Disorders | 297 |
| Neurotic Disorders, Personality Disorders, and Other Nonpsychotic Mental Disorders | 300-316 |
| Anxiety, Dissociative and Somatoform Disorders | 300 |
| Personality Disorders | 301 |
| Alcohol Dependence Syndrome | 303 |
| Drug Dependence | 304 |
| Nondependent Abuse of Drugs | 305 |
| Depressive Disorder | 311 |
| Diseases of the Nervous System and Sense Organs |  |
| Parkinson’s | 332 |
| Amyotrophic Lateral Sclerosis | 335.2 |
| Pain | 338 |
| Headache Syndromes | 339 |
| Multiple Sclerosis | 340 |
| Migraines | 346 |
| Epilepsy and Recurrent Seizures | 345 |
| Disorders of the Peripheral Nervous system | 350-359 |
| Carpal Tunnel | 354 |
| Chronic Obstructive Pulmonary Disease | 490-496 |
| Crohn’s | 555 |
| Colitis | 556 |
| Inflammatory Disease of Uterus | 615 |
| Diseases of the Musculoskeletal System and Connective Tissue |  |
| Systemic Lupus Erythematosus | 710 |
| Arthropathy Associated with Infections | 711 |
| Crystal Arthropathies | 712 |
| Arthropathy Associated with other Disorders | 713 |
| Rheumatoid Arthritis and other Inflammatory Polyarthropathies | 714 |
| Osteoarthrosis and Allied Sisorders | 715 |
| Internal derangement of Knee | 717 |
| Other Derangement of Joint | 718 |
| Other and Unspecified Disorder of Joint | 719 |
| Dorsopathies | 720-724 |
| Ankylosing Spondylitis and Other Inflammatory Spondylopathies | 720 |
| Spondylosis and Allied Disorders | 721 |
| Intervertebral Disc Disorders | 722 |
| Other Disorders of Cervical Region | 723 |
| Other and Unspecified Disorders of Back | 724 |
| Rheumatism, Excluding the Back | 725-729 |
| Polymyalgia Rheumatica | 725 |
| Peripheral Enthesopathies and Allied Syndromes | 726 |
| Other Disorders of Synovium, Tendon, and Bursa | 727 |
| Disorders of Muscle, Ligament, and Fascia | 728 |
| Other Disorders of Soft Tissue | 729 |
| Generalized Pain | 780.96 |
| Injury and Poisoning |  |
| Fractures | 800-829 |
| Dislocation | 830-839 |
| Sprains and Strains of Joints and Adjacent Muscles | 840-848 |
| Injury to Nerves and Spinal Cord | 950-957 |
