# Supplementary material for: Opioid use in medical cannabis authorization adult patients from 2013 to 2018: Alberta, Canada
Source: BMC Public Health. 2021 May 1;21:843. doi: 10.1186/s12889-021-10867-w (PMC8088205; doi:10.1186/s12889-021-10867-w)
Supplement: Supplementary file 2 — Additional file 2. Health Conditions and ICD-10 Codes defining Comorbidities. [file 12889_2021_10867_MOESM2_ESM.docx]

**Additional file 2: Health Conditions and ICD-10 Codes defining Comorbidities**

| **Disease/Injury** | **ICD-10 Code** | |
| --- | --- | --- |
| Neoplasms | | C00-D48 |
| Type 2 diabetes mellitus | | E11 |
| Diabetes Mellitus | | E10-E14 |
| Mental Disorders | |  |
| Alcohol Induced Mental Disorders | | F11 |
| Drug-induced Mental Disorders | | F11-F19 |
| Schizophrenic Disorders | | F20-F29 |
| Episodic Mood Disorders | | F30-F39 |
| Delusional Disorders | | F22-F24 |
| Neurotic Disorders, Personality Disorders, and Other Nonpsychotic Mental Disorders | | F40-F48 |
| Anxiety, Dissociative and Somatoform Disorders | | F40-F42 |
| Personality Disorders | | F60-F69 |
| Alcohol Dependence Syndrome | | F10.2 |
| Drug Dependence | | F11.2-F19.7 |
| Nondependent Abuse of Drugs | | F55 |
| Depressive Disorder | | F32 |
| Diseases of the Nervous System and Sense Organs | |  |
| Parkinson’s | | G20 |
| Amyotrophic Lateral Sclerosis | | G12.2 |
| Pain | | R52 |
| Headache Syndromes | | G44 |
| Multiple Sclerosis | | G35 |
| Migraines | | G43 |
| Epilepsy and Recurrent Seizures | | G40 |
| Disorders of the Peripheral Nervous system | | G60-G64 |
| Carpal Tunnel | | G56.0 |
| Chronic Obstructive Pulmonary Disease | | J44 |
| Crohn’s | | K50 |
| Colitis | | K50-K52 |
| Inflammatory Disease of Uterus | | N71 |
| Diseases of the Musculoskeletal System and Connective Tissue | |  |
| Systemic Lupus Erythematosus | | M32 |
| Arthropathy Associated with Infections | | M00-M003 |
| Crystal Arthropathies | | M11, M14.1 |
| Arthropathy Associated with other Disorders | | M14 |
| Rheumatoid Arthritis and other Inflammatory Polyarthropathies | | M06 |
| Osteoarthrosis and Allied Disorders | | M19.0-M19.2 |
| Internal derangement of Knee | | M23.9 |
| Other Derangement of Joint | | M24 |
| Other and Unspecified Disorder of Joint | | M20-M25 |
| Dorsopathies | | M40-M54 |
| Ankylosing Spondylitis and Other Inflammatory Spondylopathies | | M45 |
| Spondylosis and Allied Disorders | | M47 |
| Intervertebral Disc Disorders | | M51 |
| Other Disorders of Cervical Region | | M50 |
| Other and Unspecified Disorders of Back | | M53.8 |
| Rheumatism | | M79.0 |
| Polymyalgia Rheumatica | | M35.3 |
| Peripheral Enthesopathies and Allied Syndromes | | M77.9 |
| Other Disorders of Synovium, Tendon, and Bursa | | M65-M68 |
| Disorders of Muscle, Ligament, and Fascia | | M60-M63 |
| Other Disorders of Soft Tissue | | M70-M79 |
| Generalized Pain | | R52.9 |
| Injury and Poisoning | |  |
| Fractures | | T14.3, T02.9 |
| Dislocation | | T14.3, T03 |
| Sprains and Strains of Joints and Adjacent Muscles | | T14.3, T03, S37.8 |
| Injury to Nerves and Spinal Cord | | S14, T09.3, S34.1 |
